# Optimizing treatment of brain metastases in an era of novel systemic treatments: a single center consecutive series

**DOI:** 10.1007/s11060-023-04343-1

**Published:** 2023-06-02

**Authors:** P. van Schie, B. L. T. Rijksen, M. Bot, T. Wiersma, L. G. Merckel, D. Brandsma, A. Compter, P. C. de Witt Hamer, R. Post, G. R. Borst

**Affiliations:** 1 Department of Neurosurgery, Amsterdam UMC, Vrije Universiteit Amsterdam, De Boelelaan 1117, Amsterdam, The Netherlands; 2 Department of Neurosurgery, Amsterdam UMC location University of Amsterdam, Meibergdreef 9, Amsterdam, The Netherlands; 3 Amsterdam Neuroscience, Amsterdam, The Netherlands; 4 Cancer Center Amsterdam, Amsterdam, The Netherlands; 5 Department of Radiation Oncology, Netherlands Cancer Institute – Antoni van Leeuwenhoek, Plesmanlaan 121, 1066 CX Amsterdam, The Netherlands; 6 Department of Neurology, Netherlands Cancer Institute – Antoni van Leeuwenhoek, Plesmanlaan 121, 1066 CX Amsterdam, The Netherlands; 7 Division of Cancer Sciences, School of Medical Sciences, School of Biological Sciences, Faculty of Biology, Medicine and Health & Manchester Cancer Research Centre, Manchester Academic Health Science Centre (MAHSC), University of Manchester, Manchester, UK; 8 Departments of Clinical Oncology, The Christie NHS Foundation Trust, Manchester, UK; 9 Department of Neurosurgery, Amsterdam University Medical Centres, Location AMC, PO Box 22660, 1100 DD Amsterdam, The Netherlands; 10 Department of Radiotherapy Related Research, The Christie NHS Foundation Trust, Dept 58, Floor 2a, Room 21-2-13, Wilmslow Road, Manchester, M20 4BX UK

**Keywords:** Brain metastases, Systemic treatment, Radiotherapy

## Abstract

**Background:**

The multidisciplinary management of patients with brain metastases consists of surgical resection, radiation treatment and systemic treatment. Tailoring and timing these treatment modalities is challenging. This study presents real-world data from consecutively treated patients and assesses the impact of all treatment strategies and their relation with survival. The aim is to provide new insights to improve multidisciplinary decisions towards individualized treatment strategies in patients with brain metastases.

**Methods:**

A retrospective consecutive cohort study was performed. Patients with brain metastases were included between June 2018 and May 2020. Brain metastases of small cell lung carcinoma were excluded. Overall survival was analyzed in multivariable models.

**Results:**

676 patients were included in the study, 596 (88%) received radiotherapy, 41 (6%) awaited the effect of newly started or switched systemic treatment and 39 (6%) received best supportive care. Overall survival in the stereotactic radiotherapy group was 14 months (IQR 5–32) and 32 months (IQR 11–43) in patients who started or switched systemic treatment and initially did not receive radiotherapy. In patients with brain metastases without options for local or systemic treatment best supportive care was provided, these patients had an overall survival of 0 months (IQR 0–1). Options for systemic treatment, Karnofsky Performance Score ≥ 70 and breast cancer were prognostic for a longer overall survival, while progressive extracranial metastases and whole-brain-radiotherapy were prognostic for shorter overall survival.

**Conclusions:**

Assessing prognosis in light of systemic treatment options is crucial after the diagnosis of brain metastasis for the consideration of radiotherapy versus best supportive care.

**Graphical Abstract:**

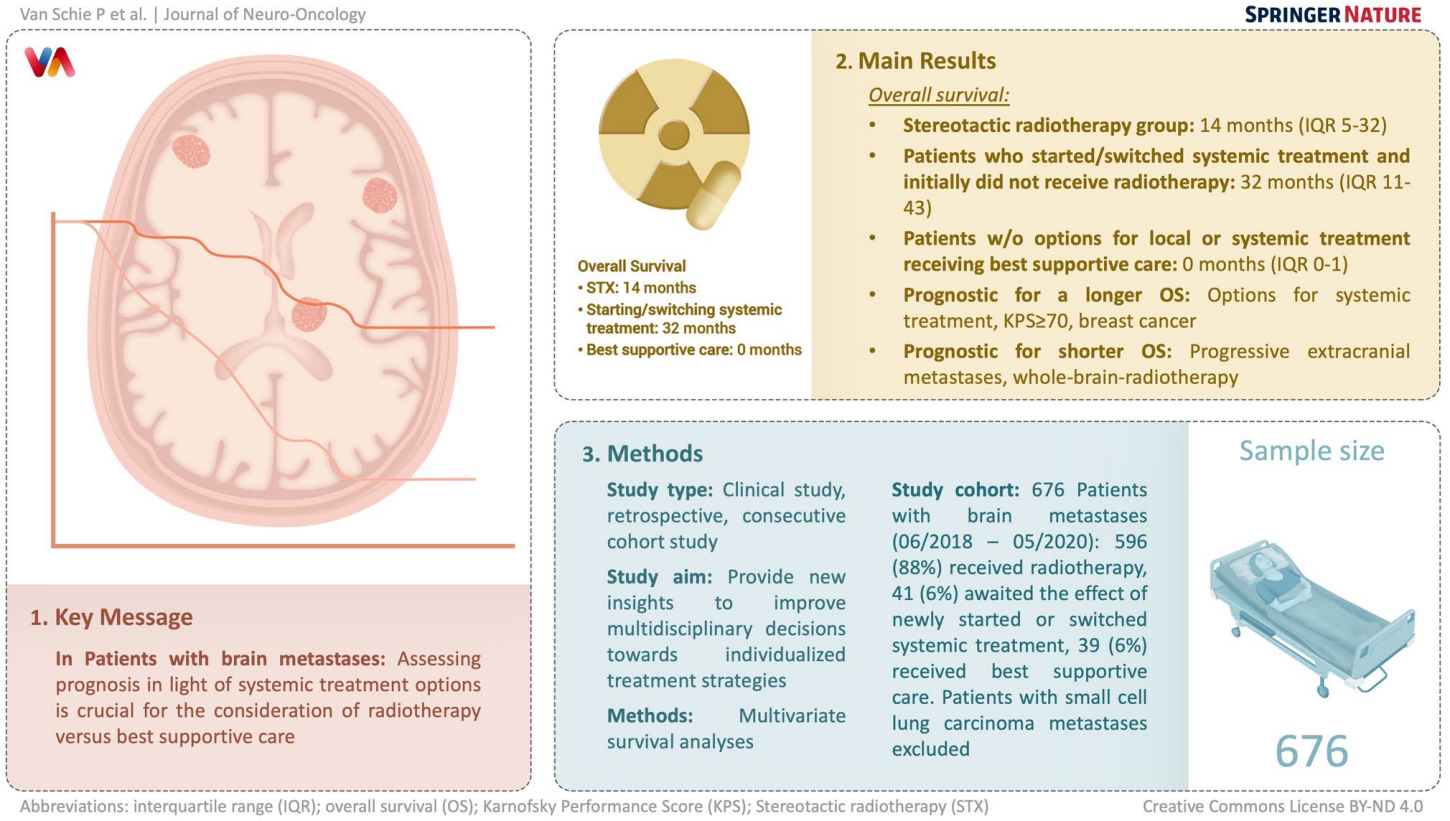

## Introduction

While novel systemic treatments such as targeted molecular treatment and immunotherapy are successfully applied to target extracranial metastases (ECM), the question whether to treat brain metastases (BM) systemically as well is a more debated field [1–3]. Intracranial metastases can respond to systemic treatment, but response depends on the agent, tumor type and the molecular driver of the tumor [4]. In addition, a wide variability of response rates, time to response and duration of response has been reported [5–7]. Furthermore, a large variety of clinical parameters need to be considered in triaging patients between systemic, local or combined treatment modalities [8, 9]. To optimize the treatment selection, the identification of the most important predictive parameters is essential. Although predictive models and guidelines for treatment selection in individual patients have been published [9–11], treatment decisions for the individual patient remain challenging.

In this study, we analyze real-world data of patients with BM consecutively treated with radiotherapy in our center in the recent era of novel systemic treatments. This study aims to evaluate the role of systemic treatment in the multidisciplinary approach and treatment decisions for patients with BM.

## Methods

### Study design and patients

A single-center, retrospective cohort study was conducted in a tertiary referral, comprehensive cancer center in the Netherlands. All patients with BM, aged 18 years or older were consecutively included in the study between June 1st 2018 and May 30th 2020. Patients with a primary, non-metastatic intracranial tumor were excluded, as were patients with BM from small cell lung carcinoma who received prophylactic cranial radiotherapy [12]. Furthermore, 55 patients were excluded, because they already received radiotherapy for the same BM earlier in the course of their disease. The institutional review board of the hospital approved the study (IRBd20-161).

### Clinical data

The following baseline characteristics were retrieved from medical charts: sex, age (dichotomized into ≤ 65 years versus > 65 years), primary tumor (NSCLC (non-small cell lung carcinoma), breast, melanoma or other), Karnofsky Performance Score (KPS) (< 70 vs. ≥ 70), status of ECM at the time BM were diagnosed (stratified into: progression, stable, regression, none present) and whether patients had previous radiotherapy for other BM. Furthermore, targeted therapy was defined as therapy against targetable mutations. These included anaplastic lymphoma kinase (ALK), epidermal growth factor receptor (EGFR) and programmed death-ligand 1 (PD-L1) in NSCLC, and BRAF in melanoma. In addition to reviewing patient files, all multidisciplinary neuro-oncology board meetings in the abovementioned period were reviewed in order to collect information about medical decision-making.

### Radiotherapy and systemic treatment parameters

#### Radiotherapy

The following items were assessed for patients undergoing stereotactic radiotherapy: number of irradiated BM, total tumor volume, and the volume of the largest lesion. These data were unavailable for patients receiving WBRT. Furthermore, the type of radiotherapy was evaluated. Patients who received stereotactic radiotherapy (SRT) in a single session were classified as ‘single fraction’, those that received radiotherapy in several consecutive sessions as ‘fractionated’ and patients who underwent radiotherapy in multiple sessions with 2–4 weeks interval as ‘staged’ [13]. Postoperative cavity SRT was classified as ‘postoperative’ and whole brain radiotherapy as ‘WBRT’.

#### Systemic treatment

The status of systemic treatment possibilities at the time of BM diagnosis was classified as follows:

1. ‘First ST’: if patients presented synchronously with their primary tumor and BM and started their first line of systemic treatment.
2. ‘Continued ST’: if the same systemic treatment was continued after BM diagnosis, in patients with metachronous BM;
3. ‘Switched ST’: if the type of systemic treatment was switched after BM diagnosis, in patients with metachronous BM;
4. ‘No ST, but options’: if patients had a metachronous BM, without ECM, without the indication for systemic treatment;
5. ‘No options for ST’: if no further options for systemic treatment were available.

### Follow-up

The primary outcome measure was overall survival (OS) which was defined as the time in months between diagnosis of BM and death, assessed using clinical reports. If clinical reports provided insufficient information, general practitioners were contacted to provide the date of death. The last follow-up was on March 22nd 2023.

### Statistical analysis

Kaplan-Meier analysis was performed to assess OS. In case patients underwent multiple radiotherapy sessions during the follow-up period, characteristics of the first radiotherapy session were used for analysis.

For statistical analysis, patients were divided into three groups, in accordance with treatment decisions made in the multidisciplinary neuro-oncology board meetings:

- Group 1: patients who received immediate radiotherapy at diagnosis of BM;
- Group 2: patients awaiting the effects of newly started or switch of systemic treatment (with ‘salvage’ RT), because intracranial response of systemic treatment was expected, which could lead to either a smaller tumor volume at subsequent radiotherapy or no radiotherapy at all;
- Group 3: patients who received no local treatment, but started best supportive care after diagnosis of BM.

The comparison between subgroups was made with the Chi-Square test or Fisher exact test. Univariate analyses were performed with log-rank test for categorical data. The Cox proportional hazards model was used in continuous data. Stepwise forward likelihood ratio multivariate analyses including all statistically significant predictors of the univariate analysis were included to assess the prognostic value of different variables using the Cox proportional hazard. For the group of patients receiving best supportive care, only descriptive statistics were used. Statistical analysis was performed using IBM SPSS Statistics for Windows, version 26 (IBM Corp., Armonk, N.Y., USA).

## Results

### Patient and treatment characteristics

**Fig. 1:**
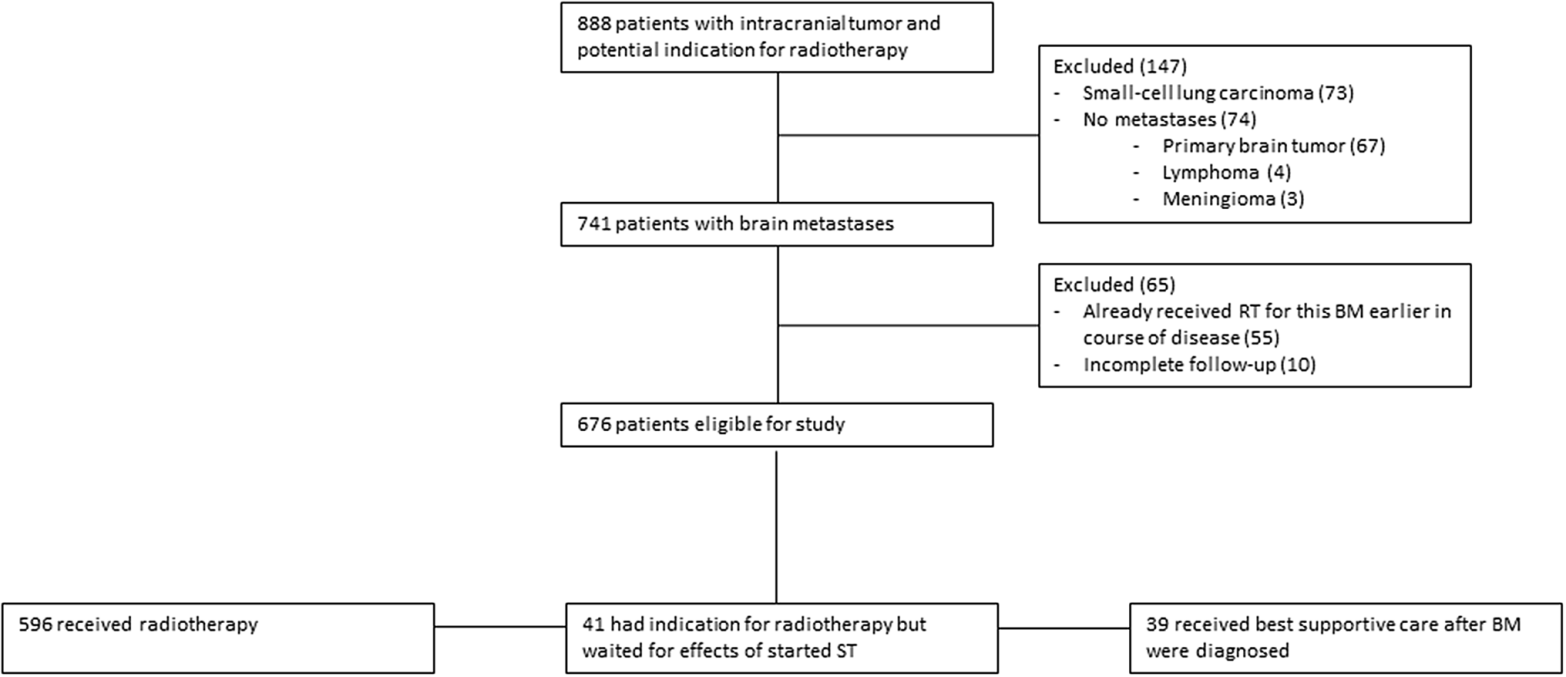
Study design. Of the 65 patients excluded between step 2 and 3 55 had a session of RT for BM before in the course of their disease, and 10 had an incomplete follow-up

676 consecutively diagnosed BM patients were included (Fig. 1; Table 1) with a median follow-up of 43 months (IQR 36–48). 101 (15%) patients had breast cancer, 322 (48%) NSCLC, 131 (19%) melanoma and 122 (18%) had other primary tumor types. Best supportive care only was given in 39 (6%) patients.

**Table 1:** Clinical characteristics of patients that received radiotherapy, measured immediately before stereotactic radiosurgery, with univariate and multivariate analysis of hazard ratios of overall survival

Variable	All	Univariate			P-value	Multivariate		P-value
	N(%)	HR		95% CI		HR	95% CI	
Overall	596 (100)							
Age, y								
< 65	407 (68)	Ref				Ref		
≥ 65	189 (32)	1.3		1.1–1.6	0.008	1.3	1.0–1.6	0.025
Gender								
Male	265 (45)	Ref						
Female	331 (55)	1.0		0.8–1.2	0.977			
Primary cancer								
NSCLC	286 (48)	Ref				Ref		
Breast	92 (15)	0.8		0.6–1.1	0.183	0.7	0.5–0.9	0.020
Melanoma	97 (16)	0.6		0.5–0.8	< 0.001	0.8	0.6-1.0	0.061
Other	121 (20)	1.2		1.0–1.7	0.067	1.0	0.7–1.2	0.762
KPS								
≥ 70	481 (81)	Ref				Ref		
< 70	115 (19)	2.5		2.0–3.1	< 0.001	1.8	1.4–2.4	< 0.001
ECM status								
None	155(26)	Ref				Ref		
Progression	246 (41)	2.0		1.6–2.5	< 0.001	1.9	1.4–2.7	< 0.001
Stable	145 (24)	1.4		1.1–1.9	0.008	1.2	0.9–1.7	0.229
Remission	50 (8)	0.7		0.7–1.5	0.958	1.4	0.9–2.3	0.124
Systemic treatment before BM diagnosis								
Yes	399 (67)	Ref				Ref		
No	180 (30)	1.2		1.0–1.5	0.028	1.1	0.7–1.9	0.728
Unknown	17 (3)	–		–	–	–	–	–
Indication systemic treatment after BM diagnosis								
First ST	131 (22)	Ref				Ref		
Continued ST	62 (10)	0.8		0.5–1.1	0.162	1.1	0.7–1.8	0.708
Switched ST	190 (32)	1.4		1.1–1.7	0.018	1.7	1.3–2.3	< 0.001
No ST (but options)	103 (17)	1.0		0.8–1.4	0.924	1.4	0.8–2.5	0.296
No options for ST	33 (6)	4.4		2.9–6.6	< 0.001	4.7	2.9–7.7	< 0.001
Unknown	77 (13)	–				–		
Prior cranial radiotherapy								
None	511 (86)	Ref						
WBRT	36 (6)	0.7		0.5–1.1	0.134			
SRT	49 (8)	0.7		0.5–1.0	0.056			
Type of cranial radiotherapy								
Single fraction	299 (50)	Ref				Ref		
Fractionated	44 (7)	1.9		1.3–2.6	< 0.001	1.6	1.1–2.4	0.010
Staged	88 (15)	1.5		1.2-2.0	0.001	1.4	1.0–1.9	0.011
Postoperative	70 (12)	0.9		0.7–1.3	0.733	1.0	0.7–1.4	0.865
WBRT	95 (16)	3.5		2.7–4.5	< 0.001	2.8	2.2–3.9	< 0.001

*BM* brain metastases,* ECM* extracranial metastases,* KPS* Karnofsky performance Score,* SRT* stereotactic radiotherapy,* ST* systemic treatment,* WBRT* whole brain radiotherapy

### Patient and treatment characteristics of patients who received immediate radiotherapy at diagnosis of BM

A total of 596 patients received radiotherapy, of which 501 (84%) patients received SRT and 95 (16%) patients WBRT. Median OS in the group receiving SRT was 14 months (IQR 5–32) versus 2 months (IQR 1–6) in case of WBRT. Median age in the group receiving radiotherapy was 60 years (IQR 51–67), and 331 (55%) patients were female (Table 1).

Of NSCLC patients, 146 (58%) of NSCLC patients had a targetable mutation, as had 62 (65%) melanoma patients. Of these patients, 95 (65%) with NSCLC received or had an option to start targeted therapy after BM diagnosis, as did 24 (39%) patients with melanoma. Furthermore, 25 of 62 (40%) of patients with melanoma and targetable mutations received immunotherapy.

**Fig. 2:**
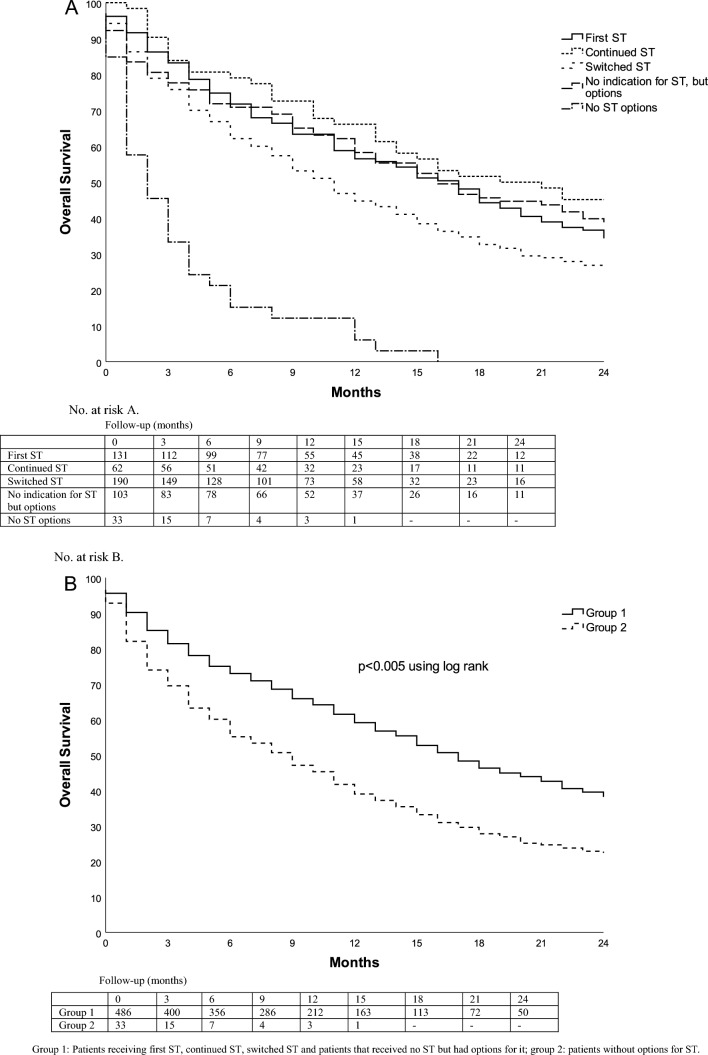
Kaplan-Meier Curves for Overall Survival according to remaining options for systemic treatment. **A** Individual groups of different options for systemic treatment. Median OS for the group receiving the first ST was 17 months (95% CI 13–20); median OS in the group that continued ST was 19 months (95% CI 7–31); median OS in the group that switched ST was 11 months (95% CI 9–13); median OS in the group that received no ST bur had options was 16 months (95% CI 11–21); median OS in the group that had no options for ST was 2 months (95% CI 1–3). **B** All patients receiving systemic treatment, or having options for it, versus patients without options for systemic treatment. Median OS for group 1 was 14 months (95% CI 12–16); median OS for group 2 was 2 months (95% CI 1–3)

Differences in OS between groups with different systemic treatment possibilities are shown in (Fig. 2). Patients who switched systemic treatment or had no options for it, had a shorter OS than patients that started or continued systemic treatment, or received none but had options for it.

Factors associated with a significant shorter OS in the univariate analysis are shown in Table 1. The multivariate analysis identified six clinical factors associated with shorter OS: KPS < 70, progressive ECM, switched systemic treatment or no options for systemic treatment and fractionated or WBRT as radiotherapeutical modality (Table 1). Patients without options for systemic treatment (HR 4.7, 95% CI 2.9–7.7) had a shorter OS compared to patients that received systemic treatment or had options for it. Patients receiving WBRT also had a shorter OS compared to patients receiving single fraction SRT (HR 2.8, 95% CI 2.2–3.9). Breast cancer as primary tumor was associated with a longer OS (HR 0.7, 95% CI 0.5–0.9).

In patients undergoing SRT, no significant correlation was found between number of BM and OS. However, total volume of BM being either between 1 and 15 cm^3^ (HR 1.3, 95% CI 1.0–1.8) and > 30 cm^3^ (HR 2.3 (1.3–4.0), were associated with a shorter OS (Table 2).

**Table 2:** Volumetric characteristics of the 501 patients that received SRT, measured at the last imaging before radiotherapy commenced

Variable	All	Univariate			P-value	Multivariate		P-value
	N(%)	HR		95% CI		HR	95% CI	
Overall	501 (100)							
Number of BM								
1	204 (41)	Ref						
2–4	189 (38)	1.1		0.8–1.3	0.605			
5–9	67 (13)	1.3		1.0–1.8	0.066			
≥ 10	41 (8)	1.3		0.9–2.0	0.140			
Total BM volume								
< 1 cm^3^	83 (17)	Ref				Ref		
1–15 cm^3^	298 (59)	1.3		1.0–1.8	0.043	1.3	1.0-1.8	0.043
15–30 cm^3^	68 (14)	1.4		0.9–2.1	0.061	1.4	0.9–2.1	0.105
> 30 cm^3^	52 (10)	2.4		1.6–3.5	< 0.001	2.3	1.3-4.0	0.005
Volume of largest tumor								
≤ 20 cm^3^	432 (86)	Ref				Ref		
> 20 cm^3^	69 (14)	1.5		1.2–2.0	0.002	1.1	0.7–1.7	0.831

*BM*  brain metastases

### Patient and treatment characteristics of patients that awaited the effects of newly started or switch of systemic treatment (with ‘salvage’ RT)

No local treatment was given initially for 41 (6%) patients with BM. 38 patients (93%).

started or switched systemic treatment with a potential intracranial response. Three patients (7%) did not receive any systemic treatment, although there were remaining available options (Table 3).

**Table 3:** Prognostic factors for survival < or ≥ 3 months for patients who awaited start of radiotherapy (= 41)

	Survival < 3 months N(%)	Survival ≥ 3 months N(%)	P-value
Total	4 (10)	37 (90)	-
Age, y			
<65	3 (75)	31 (84)	0.672
≥65	1 (25)	6 (16)	
KPS			
≥70	3 (75)	37 (100)	0.039
<70	1 (25)	0	
Primary cancer			
Lung	2(50)	11(30)	0.526
Breast	0	4 (11)	
Melanoma	2 (50)	22 (59)	
Other	0	0	
ECM status			
None	0	9(24)	0.225
Progression	2 (50)	7 (19)	
Stable	2 (50)	15 (41)	
Remission	0	6 (16)	
Any RT after MOB			
Yes	4 (100)	22 (59)	0.049
No	0	15 (41)	
Indication ST			
First ST	1 (25)	12 (29)	0.540
Continued ST	0	8 (19)	
Switched ST	3 (75)	19(43)	
No ST, but options	0	2 (5)	
Patient refused	0	1 (2)	

*ECM* extracranial metastases,* KPS* karnofsky performance score,* ST* systemic treatment,* MOB* multidisciplinary oncology board meeting

Fisher exact/chi square test (using likelihood ratio)

*1 patient refused any form of treatment, 1 patient wanted to wait for eventual growth before starting treatment, and 1 patient had long remission of a small cerebral spot, which was eventually treated with radiotherapy after 3 months (when the follow up scan showed growth)

Median survival of the total group was 32 months (IQR 11–43). Four patients (10%) died within 3 months, 37 (90%) survived longer than three months. Of the four patients that died within 3 months, 1 patient had rapid cerebral progression leading to coma, 1 patient had cerebral and leptomeningeal progression leading to severe epileptic seizures, 1 patient had rapid progression of ECM and 1 patient opted for best supportive care because of severe pain due to progression ECM.

8 (61%) of 13 patients with NSCLC had targetable mutations and all 8 (100%) patients received targeted therapy. 20 (83%) of 24 patients with melanoma had targetable mutations, 12 (60%) received targeted therapy.

27 (66%) of the 41 patients did receive radiotherapy in the further course of the disease (4 (10%) within three months, 23 (56%) after three months). The median time between the decision to wait for the effects of systemic treatment and the start of eventual SRT was 4 months (IQR 2–7).

### Patients with best supportive care only

39 (6%) patients received best supportive care directly after the BM diagnosis. All patients had a KPS < 70, and 28 patients (72%) had progressive ECM. 11 (28%) patients presented with synchronous BM and therefore had no systemic treatment before, 12 patients (31%) had possibilities for a switch in systemic treatment, 7 (18%) had no systemic options. In 7 (18%) patients clinical condition was too poor for systemic treatment and 2 (5%) patients refused systemic treatment. The median OS was 0 months (IQR 0–1).

## Discussion

In this study, we present the OS of a consecutive cohort of patients with BM presented in our center from June 2018 to May 2020 and treated with radiotherapy and/or systemic therapy.

OS was 14 months (IQR 5–32) for patients receiving SRT immediately after BM diagnosis and 2 months (IQR 1–6) in patients whom received WBRT. In the group that awaited the effect of systemic treatment on BM, OS was 32 months (IQR 11–43).

Our study confirms the earlier stated correlation of clinical predictors such as age, KPS [10], type of primary tumor [11] and total tumor volume of BM [14] for prognosis in patients with BM. In line with previous studies, no correlation between the number of BM and survival was found [15, 16]. The patient group treated directly with radiotherapy after BM diagnosis consisted of patients in whom deferral of radiotherapy was not preferred. Reasons for this direct treatment with RT after BM diagnosis included symptomatic BM, large tumor volume at diagnosis and lack of systemic treatment options with a high cerebral response rate.

For half of patients that awaited the intracranial response of recently started or switched systematic treatment, SRT was administered within 4 months. In NSCLC and melanoma patients that awaited the effect of systemic treatment, 28 of 37 patients (76%) had a targetable mutation. In the group that received radiotherapy directly after BM diagnosis, 208 of 383 (54%) patients with NSCLC and melanoma had a targetable mutation.

Also, a larger proportion received, or had options for, targeted therapy. Systemic treatment options for BM is a prognostic factor besides KPS, primary tumor type and status of extracranial metastases, and should be considered in treatment decisions for patients with BM. The expected intracranial response of the systemic treatment and its expected duration determine whether BM are treated with SRT only, a combination of SRT followed by a (switched) systemic treatment, systemic treatment only, or delayed SRT after intracranial response on systemic therapy.

The survival of the cohort that received WBRT was poor, half of the patients died within 2 months. Whether WBRT should have a place in treating palliative patients with BM has been debated before [18], considering the (sub)acute side-effects of this treatment in a patient group with a poor prognosis [19]. Moreover, half of the patients that received best supportive care after the BM diagnosis died within 1 month. All patients had a KPS < 70, 72% had also progressive ECM and 36% did not have further systemic treatment options or had a clinical condition that did not allow systemic treatment. This further underlines the important role of performance score, extracranial disease status and systemic treatment options in prognosis of patients with BM.

Our study stresses the important role of systemic treatment for individualizing treatment strategy in patients with BM. Firstly, systemic treatment with an intracranial response can postpone or prevent local treatment in patients that newly started or recently switched systemic treatment.

In our study, the time between the decision to await systemic treatment effect and treat eventually with radiotherapy was close to 4 months for half of the patients. In the group of patients with more than 3 months survival, 41% did not receive radiotherapy at all after the diagnosis of BM. Systemic treatment can be initially preferred over radiotherapy in patients with large tumor volume or high number of BM and stable extracranial disease. A treatment strategy could be to start with systemic treatment (with a potential to have intracranial activity) to reduce the volume or number of BM and to give subsequent radiotherapy at the time of progression.

Secondly, in case patients are in need for systemic treatment due to extracranial disease and a low likelihood of intracranial response is to be expected, the choice of local treatment modality is important in the overall treatment strategy. Radiotherapy in patients who are not in direct need of surgery (i.e. due to medically urgent situations or neurological symptoms that did not respond well to steroids) can expedite the start of systemic treatment compared to surgery and this could lead to survival benefits. In our data, this resulted in a relatively low percentage (12%) of patients that underwent surgery as a primary local treatment. This hypothesis stresses that more real world data is needed for optimizing the treatment strategies for individual patients including analysis of survival, tumor response, toxicity and quality of life. More insights have become available to estimate the intracranial responses and ability to defer or prevent local therapy [20–25], which is also acknowledged in the recently published ASCO-SNO-ASTRO and EANO Guidelines [9, 26]. Our study shows real-world data to support these findings. Secondly, the results of our study suggest that systemic treatment should not merely be considered as a treatment option, but should be taken into consideration as a prognostic factor for survival. In other words, for patients without systemic treatment options, local treatment like radiotherapy should be questioned. Recently, a prediction model was published, taking available types of systemic treatment into consideration, along with known clinical parameters such as age, primary tumor and KPS [27]. Volume of the largest lesion or total tumor volume were not integrated in this model. Based on our results, the availability of options for systemic treatment and total tumor volume should be added to a future prognostic prediction model.

### Strengths and limitations

This study presents a large, consecutive cohort of all patients in our center diagnosed with BM, regardless of treatment after the diagnosis and the status of their systemic treatment. To our knowledge, this is the largest cohort of patients with BM in which the systemic treatment status is included.

Several limitations should be addressed. Firstly, our series was obtained in a single, tertiary referral, comprehensive cancer center, which could have affected the patient and treatment characteristics and OS. All patients underwent intracranial follow-up in our center, but a subset of patients underwent systemic follow-up in the referring hospital. Although we were able to retrieve dates of death in all patients, it was not always possible to make a robust evaluation whether the cause of death was caused by either progressive intracranial or extracranial disease, or both.

Since this study was a retrospective cohort study, we could not accommodate for the differences in assessing clinical features such as KPS between different physicians.

In conclusion, this study shows that in a selected group of patients with BM, that started a new line of systemic treatment with an expected significant intracranial response rate, the response on BM could be awaited, and SRT deferred. There is a limited role for radiotherapy in patients with progressive ECM and no systemic treatment options. Furthermore, our results endorse the important role that (new) systemic treatment options have on OS. We recommend that the availability of systemic treatment options should always be considered in determining treatment strategies in patients with BM.

## Data Availability

The datasets generated during and/or analysed during the current study are available from the corresponding author on reasonable request.

## Abbreviations

BM: Brain metastases
ECM: Extracranial metastases
KPS: Karnofsky performance score
ST: Systemic treatment
SRT: Stereotactic radiotherapy
WBRT: Whole brain radiotherapy
